# Supplementary material for: Patterns of Intron Gain and Loss in Fungi
Source: PLoS Biol. 2004 Nov 30;2(12):e422. doi: 10.1371/journal.pbio.0020422 (PMC532390; doi:10.1371/journal.pbio.0020422)
Supplement: Table S1 — Also available at http://genes.mit.edu/NielsenEtAl/. (4.3 MB ZIP). [file pbio.0020422.st001.zip › NielsenEtAl/html/188.html]

AN4769.1.NCU01985.1.MG02643.1.FG08875.1


```
 CLUSTAL W (1.82) Multiple Sequence Alignments - Introns Inserted


Sequence 1: NCU01985.1	573 aa
Sequence 2: MG02643.1	547 aa
Sequence 3: FG08875.1	614 aa
Sequence 4: AN4769.1	574 aa
Alignment Length: 615 aa
Number Identitical Residues: 360 aa
Alignment Score (without introns) 16933


MG02643.1 	MANPPHGGVLKDLLARDAPRNAELAAEAETLPALLLTERQLCDLELIITGGFSPLEG~FM
NCU01985.1	MSNPPHGGVLKDLIARDLPRHAELEAEAETLPALLLSERQLCDLELILNGGFSPLEG1FM
FG08875.1 	MANTPHGGVLKDLFARDLPRQSELEAEAQKLPALTLSERHLCDLELILNGGFSPLEG1FL
AN4769.1  	MANTPHGGVLKDLIARDAPRHDQLEAEAATLPSIVLTERQLCDLELIMNGGFSPLEG1FM
          	*:*.*********:*** **: :* *** .**:: *:**:*******:.******** *:

MG02643.1 	TEKDYNG~VVKENRLTDGALFSMPITLDLSQQTIDENGVKPGARITLRDFRDDRNLTILT
NCU01985.1	NQEDYND2VVKENRLASGLLFSMPITLDVSEETISELGLKAGARITLRDFRDDRNLAILT
FG08875.1 	TEKDYNG2VVENNRLADGALFSMPINLDVNQAEIDQLGLKAGARVTLRDFRDDRNLAILT
AN4769.1  	NQKDYDG2VVAESRLADGNLFSMPITLDASKAVIEQAGLKPGSRVTLRDFRDDRNLAILT
          	.::**:. ** :.**:.* ******.** .:  *.: *:*.*:*:***********:***

MG02643.1 	VEDVYRPDK~QLEAKEVFGGDPE-HPAVNYLFNTAGEFYVGGKLEAVNRLQHYDF0T---
NCU01985.1	VDDVYKPDK2ALEAKEVFGGDEE-HPAVKFLYETAKEYYVGGKLEAVNKLQHYDF~VDLR
FG08875.1 	VEDIYRPDK2VNEAKKVFGSDDDTHPGVKYLFDTAKEFYVGGKLEAINRLEHYDF~LDLR
AN4769.1  	IDDIYRPDK2EKEAKLVFGGDPE-HPAIKYLNTKVEEYYIGGKLEAVNKLNHYDY~VGLR
          	::*:*:***   *** ***.* : **.:::*  .. *:*:******:*:*:***:  .  

MG02643.1 	-~----------------------R~NPMHRAHRELTVRAARSHHANVLIHPVVGLTKPG
NCU01985.1	Y1SPAEIRTHFDKLGWSRVVAFQTR~NPMHRAHRELTVRAARSHHANVLIHPVVGLTKPG
FG08875.1 	F1TPSELRAHFNKLGWQKVVAFQTR~NPMHRAHRELTVRAARSQQANVLIQPVVGLTKPG
AN4769.1  	Y1TPAELRIHFDKLGWTRVVAFQTR2NPMHRAHRELTVRAARARQANVLIHPVVGLTKPG
          	  :.:.     .. .     : .:* ****************:::*****:*********

MG02643.1 	DIDHFTRVRVYKALLPRYPNGMAVLGLLPLAMRMGGPREAIWHAIIRKNHGATHFIVGRD
NCU01985.1	DIDHFTRVRVYKALLPRYPNGMAVLGLLPLAMRMGGPREAIWHAIIRKNHGATHFIVGRD
FG08875.1 	DIDHFTRVRVYKALLPRYPNGMAALALLPLAMRMGGPREALWHAIIRKNHGATHFIVGRD
AN4769.1  	DIDHFTRVRAYQALLPRYPNGMAALALLPLAMRMGGPREAVWHAIIRKNHGATHFIVGRD
          	*********.*:***********.*.**************:*******************

MG02643.1 	HAGPGKNSAGVDFYGPYDAQYAVEKYKDELGIEVVPFQMMTYLPDSDEYAPVDTIEKGVR
NCU01985.1	HAGPGKNSKGVDFYGPYDAQYAVEKYRDELGIEVVPFQMMTYLPDSDEYAPVDQIPKGVR
FG08875.1 	HAGPGKNKQGKDHYGPYDAQVLVQEHQEELGIKMVEFQAMIYLPDSDEYLPINEIPEGTR
AN4769.1  	HAGPGKNSKGQEFYGPYDAQHAVEKYRAELGIEVVEFQQVTYLPDTDEYMPKDEVPAGTK
          	*******. * :.*******  *:::: ****::* ** : ****:*** * : :  *.:

MG02643.1 	TLNISGTELRSRLRSGRDIPEWFSYPEVVKVLRESHPPRSQQGFTIFLTGYQNSGKDQIA
NCU01985.1	TLNISGTELRARLRSGREIPEWFSYPEVVKVLRESHPPRSQQGFTVLFTGYPNSGKDQIA
FG08875.1 	TLNISGTELRHRLRTGKDIPEWFSYPEVVKVLREENPLPAEKGFTVFMTGYQNSGKDQIA
AN4769.1  	TLDISGTELRNRLRTGAHIPEWFSYPEVVKILRESSPPRALQGFTIFLTGYMNSGKDAIA
          	**:******* ***:* .************:***. *  : :***:::*** ***** **

MG02643.1 	RALQVTLNQQGGRSVSMLLGETVRSELSSELGFSREDRDKNIGRIAFVASELTRSGAAVI
NCU01985.1	RALQVTLNQQGGRSVSLFLGENIRHELSSELGYNREDRDKNIARIAFVASELTRSGAAVI
FG08875.1 	RALQATLNQGGGRPVSMLLGENVRHELSPELGFTRKDRDLNIQRIAFVASELTKAGAAVI
AN4769.1  	RALQVTLNQQGGRSVTLLLGDTVRHELSSELGFSREDRHTNVQRIAFVAGELTRAGAAVI
          	****.**** ***.*:::**:.:* ***.***:.*:**. *: ******.***::*****

MG02643.1 	AAPIAPFEASRQHARELVEKHG-DFYLVWVNTPLEHCEKTDKRGIYKRARAGEIKGFTGV
NCU01985.1	AAPIAPFEKARQNARELVEKYG-DFYLVHVATPLEYCEKTDKRGIYAKARAGEIENFTGV
FG08875.1 	AAPIAPFEDARKAARDLVEKSG-PFFLVHVATPLEYCEKTDRKGVYAAARNGEIKNFTGV
AN4769.1  	AAPIAPYEHSRKAAREAVQSTGGSFFLVHVNTPLEYCEATDKRGIYAKARRGEIKGFTGV
          	******:* :*: **: *:. *. *:** * ****:** **::*:*  ** ***:.****

MG02643.1 	DDPYENPVKPDLTVDVSKQSVRSIVHQIVLTLESKGLLDRL~------------------
NCU01985.1	NDPYETPAKPDLVVDCEKQSVRSIVHQIILLLESQGLLDRF~------------------
FG08875.1 	DDPYEAPAKPDLVVDLEKQNVRSIVHQIVLLLESNGLLDRL2ARINGIGVTLNEMDKEKM
AN4769.1  	DDPYEAPTNANLVVDVSKQSVRSIVHEIILMLESEGYFERL~------------------
          	:**** *.:.:*.** .**.******:*:* ***:* ::*:                   

MG02643.1 	----------------------
NCU01985.1	----------------------
FG08875.1 	GKCDELHCIALLVYLDDEAVSG
AN4769.1  	----------------------
          	
```
